# Efficacy and safety of rilpivirine-based regimens in treatment-experienced HIV-1 infected patients: a prospective cohort study

**DOI:** 10.7448/IAS.17.4.19796

**Published:** 2014-11-02

**Authors:** Sandrine Gazaignes, Matthieu Resche-Rigon, Chloe Yang, Caroline Gatey, Anne-Lise Munier, Kristell Desseaux, Willy Rozenbaum, Jean-Michel Molina

**Affiliations:** 1 Infectious Disease Unit, Saint-Louis Hospital, Paris, France; 2 Biostatistics Department, Saint-Louis Hospital, Paris, France; 3 Medical Department, University of Toronto, Toronto, Canada

## Abstract

**Introduction:**

Rilpivirine (RPV) is a new once-daily, non-nucleoside, reverse transcriptase inhibitor (NNRTI). In treatment-naïve patients, RPV has shown non-inferior antiviral activity to efavirenz but data in treatment-experienced patients are more limited. We assessed the efficacy and safety of RPV in treatment-experienced patients switching to a RPV-based regimen.

**Methods:**

Between September 2012 and June 2013, all antiretroviral therapy (ART) experienced HIV-1 infected patients with a plasma HIV-RNA level <50 cp/mL, and switching to a RPV-based regimen, were enrolled in this prospective monocentric cohort study. Clinical and laboratory data were collected every 3 months to assess safety and efficacy. The primary endpoint was the proportion of patients with virologic success (HIV-RNA load <50 cp/mL) at 12 months using the FDA snapshot algorithm.

**Results:**

A total of 281 patients (76% male, median age: 47 years, 56% MSM) were enrolled in this study. Median lymphocyte CD4 count at baseline was 640/mm^3^. Patients have received ART for a median of 7 years and viral replication was fully suppressed for a median of 3 years. Before the switch, 39% patients were treated with NNRTI, 52% with protease inhibitor and 7% with integrase inhibitor-based regimens. Reasons for switch were simplification (176 cases), adverse events (AEs) (93 cases) and others (12 cases). At month 12 (database frozen on June 2014) in the snapshot analysis, 56% of patients met virologic success, 5% experienced virologic failure (n=14) and 39% had no data in the window period. In the LOCF analysis (using data from the previous available visit before month 12), 89% patients were suppressed, 5% had virologic failure and 6% had no data. Genotypic resistance analysis was performed in 7/14 patients at the time of virologic failure (3 of whom had previous NRTI/NNRTI resistance-associated mutations (RAMs)), and new NNRTI and NRTI RAMs emerged in 4 patients. RPV-based regimen was generally well tolerated and only 6% of patients discontinued treatment for AEs. Grade 2–4 treatment-related AEs occurred in 39 patients: 6 had rashes, 13 neuropsychiatric symptoms, 10 GI symptoms, 10 biological abnormalities. At month 12, significant changes from baseline were seen for total and LDL cholesterol (−0.5 and −0.28 mmol/L, respectively, p<0.05 for both), and plasma creatinine (+5.8 µmol/L, p<10–4).

**Conclusions:**

In patients fully suppressed on ART, switching to a RPV-based regimen in clinical practice was well tolerated and associated with few virologic failures.

